# Pharmacokinetics, tissue distribution, and plasma protein binding rate of curcumol in rats using liquid chromatography tandem mass spectrometry

**DOI:** 10.3389/fphar.2022.1036732

**Published:** 2022-11-30

**Authors:** Zhaoxu Yang, Qingmei Sun, Sheng Wang, Bingbing Tang, Chenxing Yuan, Yue Wu, Jiabin Dai, Chen Yang, Lingkun Wang, Qian Zhou, Jincheng Wang, Qinjie Weng

**Affiliations:** ^1^ Zhejiang Province Key Laboratory of Anti-Cancer Drug Research, Center for Drug Safety Evaluation and Research, College of Pharmaceutical Sciences, Zhejiang University, Hangzhou, China; ^2^ Zhejiang Province Key Laboratory of Anti-Cancer Drug Research, College of Pharmaceutical Sciences, Zhejiang University, Hangzhou, China; ^3^ College of Pharmaceutical Sciences, Hangzhou Institute of Innovative Medicine, Zhejiang University, Hangzhou, China; ^4^ Department of Pharmacy, Hangzhou Medical College, Hangzhou, China

**Keywords:** curcumol, UHPLC-MS/MS, pharmacokinetics, protein binding rate, tissue distribution

## Abstract

**Objective:** Curcumol is one of the major active ingredients isolated from the traditional Chinese medicine Curcumae Rhizoma and is reported to exhibit various bioactivities, such as anti-tumor and anti-liver fibrosis effects. However, studies of curcumol pharmacokinetics and tissue distribution are currently lacking. This study aims to characterize the pharmacokinetics, tissue distribution, and protein binding rate of curcumol.

**Methods:** Pharmacokinetics properties of curcumol were investigated afte doses of 10, 40, and 80 mg/kg of curcumol for rats and a single dose of 2.0 mg/kg curcumol was given to rats *via* intravenous administration to investigate bioavailability. Tissue distribution was investigated after a single dose of 40 mg/kg of orally administered curcumol. Plasma protein binding of curcumol was studied *in vitro via* the rapid equilibrium dialysis system. Bound and unbound curcumol in rat plasma were analyzed to calculate the plasma protein binding rate. A UHPLC-MS/MS method was developed and validated to determine curcumol in rat plasma and tissues and applied to study the pharmacokinetics, tissue distribution, and plasma protein binding in rats.

**Results:** After oral administration of 10, 40, and 80 mg/kg curcumol, results indicated a rapid absorption and quick elimination of curcumol in rats. The bioavailability ranging from 9.2% to 13.1% was calculated based on the area under the curves (AUC) of oral and intravenous administration of curcumol. During tissue distribution, most organs observed a maximum concentration of curcumol within 0.5–1.0 h. A high accumulation of curcumol was found in the small intestine, colon, liver, and kidney. Moreover, high protein binding rates ranging from 85.6% to 93.4% of curcumol were observed in rat plasma.

**Conclusion:** This study characterized the pharmacokinetics, tissue distribution, and protein binding rates of curcumol in rats for the first time, which can provide a solid foundation for research into the mechanisms of curcumol’s biological function and clinical application.

## Introduction

**FIGURE 1:**
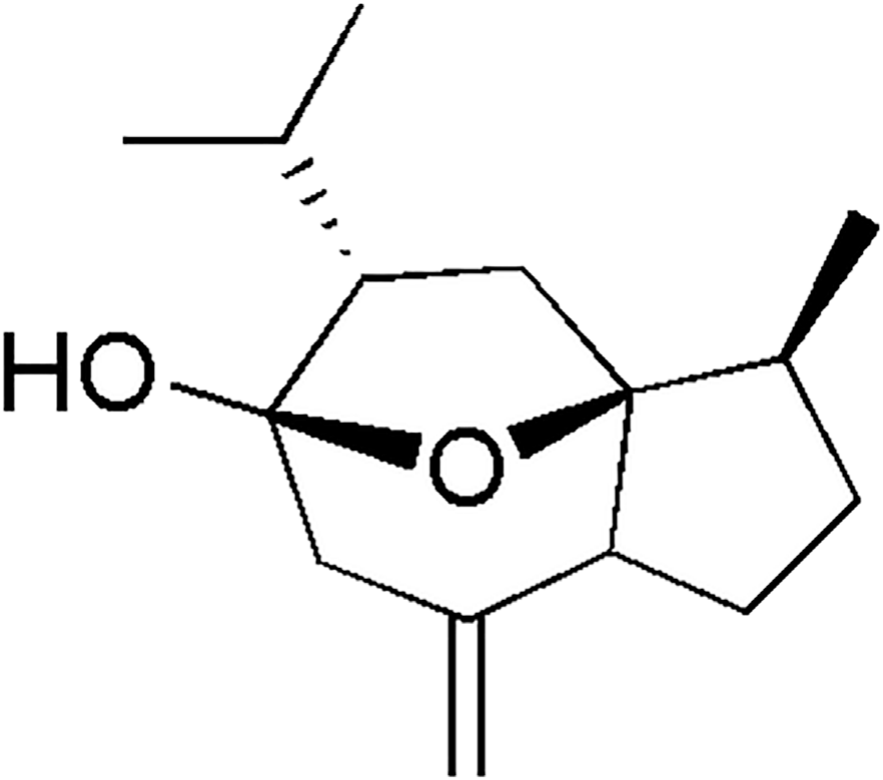
Chemical structure of curcumol.

Curcumol (Figure 1) is a natural sesquiterpenoid, isolated from the roots and rhizome of various plants, such as *C. Longa*, *C. Aromatic, and C. Zedoary.* Curcumol is one of the primary active ingredients of traditional Chinese medicine *Curcumae Rhizom*a, which has been used to treat cancer in clinical combination therapies (Aghaabbasi et al., 2020). Curcumol exhibits tumor-suppressive activity in various cancer types, such as hepatocellular carcinoma, nasopharyngeal carcinoma, lung adenocarcinoma, pancreatic, ovary, and breast cancer (Lu et al., 2012; Zuo et al., 2020; Tian et al., 2021; Fang et al., 2022; Liu, 2022). Curcumol also increased the sensitivity of cancer cells to cytotoxic antitumor drugs in the gastric and colon (Huang et al., 2020; Gao et al., 2021). Besides, the previous studies reported a potential medicinal value of curcumol in alleviating liver fibrosis by regulating liver angiogenesis (Zheng et al., 2021; Sun et al., 2022). Moreover, curcumol has shown antiviral effects by promoting IFN-β secretion (Zheng et al., 2021). These studies suggest curcumol may be a promising drug candidate for various diseases. Since numerous studies report its pharmacological potential, the absorption level and tissue distribution of curcumol are of particular interest.

Currently, several studies have been done on its pharmacokinetics and tissue distribution based on *Curcumae Rhizoma* extract or zedoary turmeric oil (Li et al., 2018; Ren et al., 2021). However, these extracts’ complex components might affect the target component’s pharmacokinetic characteristics and ADME process (Song et al., 2021). Thus, the pharmacokinetic characteristics of pure curcumol *in vivo* have yet to be illustrated clearly. A recent study has reported the concentration of curcumol in the liver after *Curcumae Rhizoma* extract oral administration. However, the components with similar structures in the extract may interfere with evaluating the individual chemical (Li et al., 2014). Therefore, it is necessary to investigate the pharmacokinetics and distribution characteristics of curcumol as an individual chemical. This study developed a rapid and sensitive UHPLC-MS/MS method to quantify curcumol in rat plasma, brain, heart, liver, spleen, lung, kidney, small intestine, and colon to characterize its pharmacokinetics, tissue distribution, and plasma binding rate. These data can serve as a foundation for its preclinical pharmacokinetic and distribution characteristics *in vivo* and support further study for curcumol clinical application.

## Materials and methods

### Reagents and materials

Curcumol (purity ≥99%) was purchased from the National Institutes for Food and Drug Control (Beijing, China), and curzerenone (purity >98%) was obtained from Weikeqi Bio-Technology Co., Ltd. (Chengdu, China). HPLC-grade methanol and acetonitrile were purchased from Merck KGaA (Darmstadt, Germany). Formic acid, ethanol, and Kolliphor HS15 were purchased from Sigma-Aldrich Corp. (St. Louis, MO, United States). Milli-Q water (18.2 MΩ cm) was obtained from a Purelab OptionS7 ultra-pure water system (ELGA LabWater, High Wycombe, United Kingdom).

### Animals

Male Sprague Dawley (SD) rats (age 6 weeks) were purchased from Zhejiang Vital River Laboratory Animal Technologies Co., Ltd. in this study. (Zhejiang, China). Animal experiments were approved by the Animal Experiment Committee of the Center for Drug Safety Evaluation and Research of Zhejiang University. All animal use and studies complied with all relevant ethical regulations and were approved by the Institutional Animal Care and Use Committee (IACUC) at Zhejiang University (permit number: IACUC-s22-019). The animals were acclimatized in a laboratory environment for 7 days before the experiment. They are kept in plastic cages for 24 h, given free pellet food and water, and put on a 12-h light/dark cycle.

### Instrumentation and conditions

The UPLC system is equipped with an ACQUITY UPLC I-Class (Waters Corp., Milford, MA, United States) and an ACQUITY UPLC BEH C8 (2.1 mm × 100 mm, 1.7 µm, Waters Corp.) for separation. The mobile phase (A) was water containing 0.1% formic acid, and the mobile phase (B) was acetonitrile containing 0.1% formic acid. The gradient elution procedure was as follows; 0.00–1.00 min: 10%B→97%B, 1.00–2.30 min:97%B, 2.30–2.31 min: 97%B→10%B, 2.31–3.00 min: 10%B with a constant flow rate of 0.400 mL/min. At this flow rate, the column was subjected to pressures approaching 9,000–10,000 psi. The injection volume was 20.0 µl, and the autosampler and column temperature were maintained at 4°C and 40°C, respectively.

**FIGURE 2:**
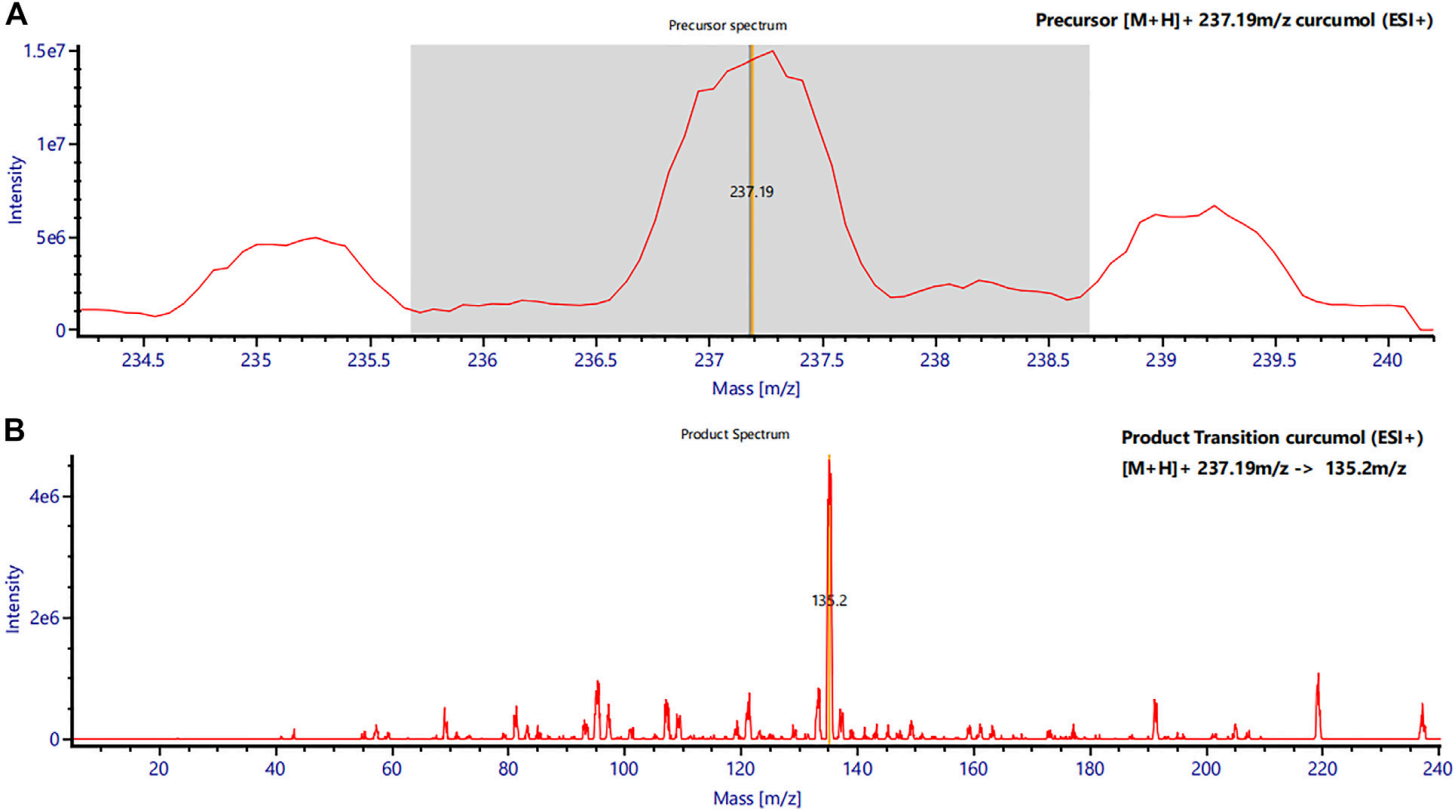
Mass spectra of curcumol in rat plasma. **(A)** Precursor mass spectrum of curcumol (m/z 237.19 [M + H]+) and **(B)** its product ions (MS/MS chromatograms; m/z 237.19 → m/z 135.2).

The mass spectrometry system consisted of a Xevo TQS triple quadrupole MS/MS (Waters Corp.) equipped with an ESI source. Multiple reaction monitoring (MRM) was selected to quantify curcumol in positive ion mode. Curcumol m/z 237.19→135.20 and IS m/z 231.19→89.13 were chosen as the MS transitions for quantification. The full scan precursor ion spectra of Curcumol (A) and product ion spectra (B) are shown in Figure 2. The optimized parameters of the instrument were obtained as follows: capillary voltage, 3.00 kV; ion source temperature, 150°C; desolvation gas temperature, 500°C; collision gas flow rate, 0.15 mL/min; desolvation gas flow rate, 1000 L/h. Data were processed using UNIFI v1.9.3 software (Waters Corp.).

### Preparation of stock solutions, calibration standards, and quality control samples

The appropriate amount of each standard substance was dissolved in methanol: water (50:50, V/V) to obtain the concentration of curcumol and IS at 1 mg/mL, respectively. Calibration curves were prepared at concentrations of 10, 20, 50, 100, 200, 500, 1,000, and 2000 ng/mL for the plasma; 5, 10, 50, 100, 200, 500, 1,000, and 2000 ng/mL for the brain, heart, liver, spleen, lung, kidney; 10, 20, 50, 100, 500, 1,000, 2000 and 5,000 ng/mL for the small intestine, and colon. The QC samples were prepared at concentrations of 30, 400, and 1,600 ng/mL for the plasma; 15, 160, and 1,600 ng/mL for the heart, liver, spleen, lung, and kidney; 30, 400, and 4,000 ng/mL for the small intestine, and colon.

### Sample preparation

About 0.20 mL blood samples were collected and centrifuged (5417R centrifuge, Eppendorf AG, Hamburg, Germany) for 10 min at 3,000 rpm to obtain the plasma. Then, 50 µl of plasma was mixed with 150 µl of acetonitrile (containing internal standard solution at a concentration of 10 ng/mL) and centrifuged at 12,000 rpm for 5 min after a vortex using a multi-tube vortex mixer (Allsheng, Hangzhou, China) for 5 min 100 µl of supernatant was taken for UHPLC-MS/MS analysis. The whole tissues were collected and washed with 0.9% normal saline, and the tissue samples (0.3 g) were homogenized in 0.9% normal saline (1: 3, w/v). Then, 75 μl of tissue homogenate mixed with 150 µl of acetonitrile (containing internal standard solution at a concentration of 10 ng/mL) was treated in the same manner as the plasma sample.

### Pharmacokinetics experiment

For the pharmacokinetics study, twenty Sprague-Dawley rats were randomly divided into four groups (*n* = 5). After weighing, single doses of 10 mg/kg, 40 mg/kg, and 80 mg/kg of curcumol were given *via* intragastric administration (i.g.) for three groups of rats, and the rest group of rats was given a single dose of 2.0 mg/kg *via* intravenous administration (i.v.). 5% ethanol and 5% Kolliphor HS15 saline were added to form a uniform emulsion for immediate administration. Rat blood was collected from the tail vein into heparinized tubes at 0 (pre-dose), 0.25, 0.50, 1.0, 2.0, 3.0, 4.0, 6.0, 8.0, 12.0, and 24 h after i.g. Dosing and at 0 (pre-dose), 0.083, 0.25, 0.50, 1.0, 2.0, 3.0, 4.0, 6.0, 8.0, 12.0 and 24 h after i.v. Dosing. After centrifugation at 3,000 rpm for 10 min at 4°C, plasma were immediately collected and stored at −80°C until analysis. See “2.5 sample preparation” for the subsequent plasma sample processing. The pharmacokinetic parameters of the study were obtained by pharmacokinetic software DAS 3.0 with non-compartment analysis.

### Tissue distribution study

In the distribution experiment, thirty SD rats were orally administered 40 mg/kg curcumol; five rats were sacrificed at 0.5, 1, 2, 4, 8, and 24 h. The tissues were collected, including the heart, liver, spleen, lung, kidney, brain, small intestine, and colon. The tissues were collected rapidly and rinsed thoroughly with normal saline (0.9%). All the tissue samples were wiped dry with filter paper and then stored at −80°C. See “2.5 sample preparation” for the subsequent tissue sample treatment and analysis.

### Plasma protein binding assay

Curcumol plasma protein binding in rat plasma was performed *in vitro.* Rat plasma was spiked with curcumol working solutions to make plasma samples at three different concentrations (100, 300, and 600 ng/mL). The bound and unbound fractions of curcumol were separated from rat plasma samples by rapid equilibrium dialysis through the RED Device Inserts (Thermo Scientific, United States). Spiked plasma samples in duplicates (300 μl) were placed in the sample chamber with 550 μl dialysis buffer placed into the buffer chamber. After incubating for 120 min at 37°C, 50 µl plasma and 50 µl buffer samples were taken from the sample chamber and the buffer chamber, respectively. Then pieces mixed with 150 µl of acetonitrile (containing internal standard solution at a concentration of 10 ng/mL) were treated and centrifuged at 12,000 rpm for 5 min after a vortex using a multi-tube vortex mixer (Allsheng, Hangzhou, China) for 5min. Supernatant was taken for UHPLC-MS/MS analysis.

## Results

### Method validation

**FIGURE 3:**
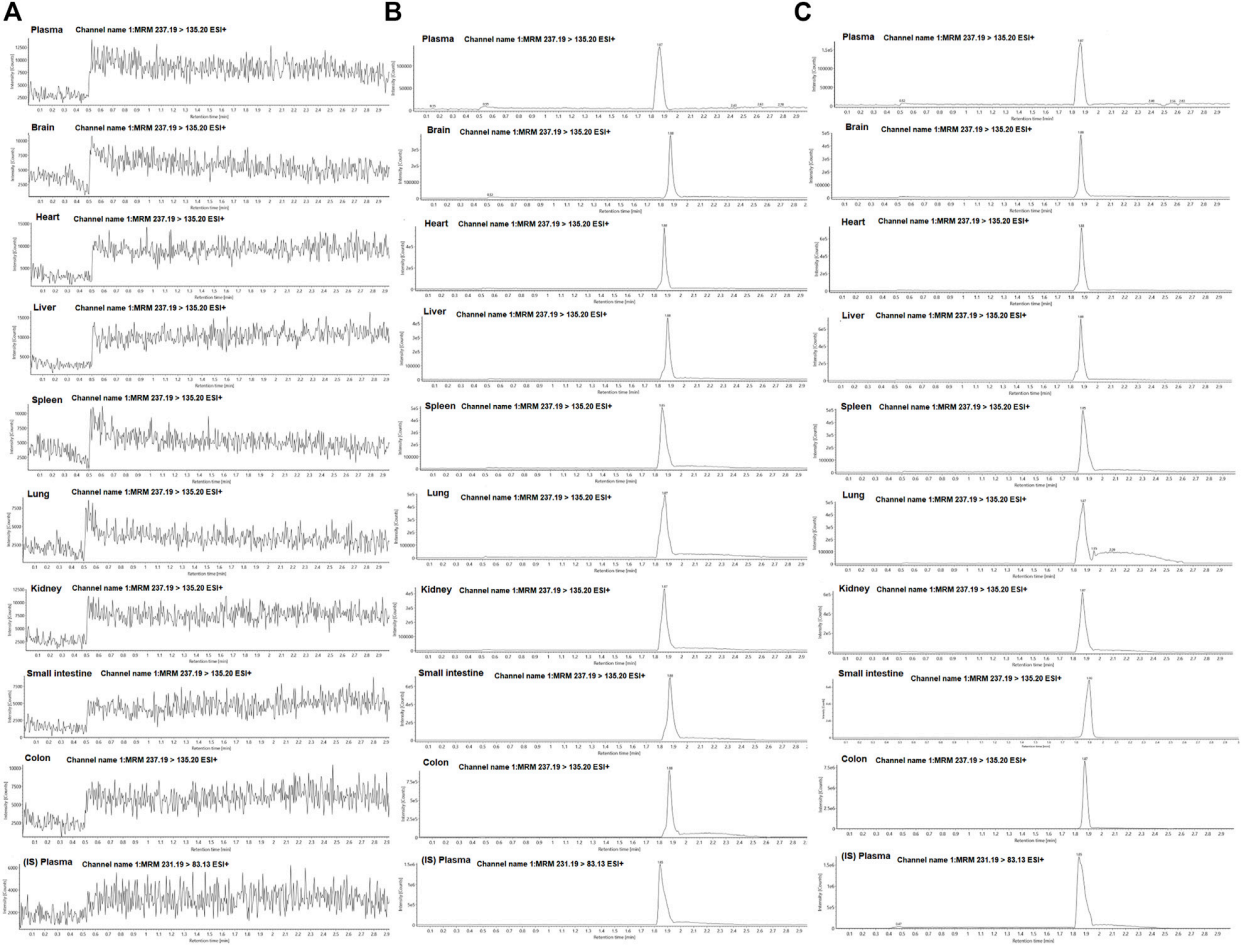
Representative MRM chromatograms of curcumol and IS in plasma and tissue samples; **(A)** blank plasma and tissue samples; **(B)** plasma and tissue samples spiked with curcumol and IS; **(C)** plasma and tissue samples collected after oral administration of curcumol.

To determine the concentration of curcumol in plasma and tissues, we established standard curves for each matrix from corresponding blank tissue and spiking different concentrations of curcumol. Representative chromatograms of curcumol in plasma and tissues of the blank, curcumol spiked with IS, and oral groups are shown in Figure 3. The retention time of curcumol and IS were 1.87 and 1.85 min, respectively, and the total analysis time was in 3 minutes. There was no significant interfering peak observed.

The calibration curves showed good linear linearity at the concentration ranges of curcumol in rat plasma and tissue homogenates (Table 1). Quality control (QC) samples at three concentration levels were determined to evaluate the precision and accuracy. The RSDs of precision ranged from 0.9% to 12.3% for the intra-day and 1.7%–10.7% for the inter-day, and the accuracy was within ±15.0% for both the intra- and inter-day (Table 2). The extraction recoveries at three levels for curcumol were more than 89.0%, and the matrix effect ranged from 90.5% to 113.5% in plasma and tissue homogenates in Table 3.

**TABLE 1:** Calibration curves, correlation coefficients (r^2^), and linear ranges of curcumol in rat plasma and tissue homogenates.

Samples	Calibration curve	r^2^	Linear range (ng/mL)
**Plasma**	Y = 0.00288X+0.0255	0.998784	10–2000
**Brain**	Y = 0.00126X+0.00158	0.998277	5–2000
**Heart**	Y = 0.00223X+0.000912	0.994109	5–2000
**Liver**	Y = 0.00217X+0.0302	0.997367	5–2000
**Spleen**	Y = 0.00218X+0.00105	0.996730	5–2000
**Lung**	Y = 0.00203X+0.00158	0.996026	5–2000
**Kidney**	Y = 0.00265X+0.0117	0.996993	5–2000
**Small intestine**	Y = 0.00107X+0.048	0.992892	10–5,000
**Colon**	Y = 0.00102X-0.00123	0.996044	10–5,000

**TABLE 2:** Accuracy and precision of curcumol in rat plasma and tissue homogenates (n = 5).

Sample	Spiked concentration (ng/mL)	Intra-day		Inter-day	
		Accuracy	Precision	Accuracy	Precision
		%	(RSD, %)	%	(RSD, %)
**Plasma**	10	105.9	8.2	100.6	7.5
	30	102.2	8.4	96.5	8.2
	400	110.9	2.0	98.3	9.8
	1,600	111.0	2.3	97.3	10.7
**Brain**	5	100.0	12.3	94.0	8.8
	15	98.0	2.1	95.7	4.5
	160	97.3	1.7	98.3	1.7
	1,600	93.6	4.4	92.5	2.9
**Heart**	5	92.4	2.5	93.8	2.8
	15	90.9	3.4	93.2	3.1
	160	88.0	2.2	99.3	8.5
	1,600	97.1	4.5	94.0	4.1
**Liver**	5	96.4	5.9	102.4	6.9
	15	99.1	8.6	104.4	6.9
	160	98.6	3.4	106.3	5.9
	1,600	100.3	3.1	98.4	5.6
**Spleen**	5	94.6	6.7	97.6	8.1
	15	104.1	7.5	102.4	6.6
	160	108.5	1.1	100.4	6.3
	1,600	101.5	5.6	96.6	6.1
**Lung**	5	96.4	7.6	97.3	7.5
	15	102	4.4	96.4	5.8
	160	107.1	1.7	99.3	8.4
	1,600	89.1	0.9	93.4	7.3
**Kidney**	5	94.9	7.3	97.3	8.0
	15	95.1	6.7	99.0	6.5
	160	94.9	4.7	101.2	8.4
	1,600	89.8	2.9	96.3	9.6
**Intestine**	10	100.6	8.6	102.0	7.4
	30	100.7	6.0	102.9	4.7
	400	111.0	2.5	108.4	4.9
	4,000	94.1	3.4	95.9	3.9
**Colon**	10	109.4	3.5	102.1	8.0
	30	103.1	6.6	105.0	5.3
	400	99.0	1.2	106.5	5.5
	4,000	90.9	3.6	96.6	5.9

**TABLE 3:** The extraction recovery and the matrix effect of curcumol in rat plasma and tissue homogenates (n = 5).

Sample	Spiked concentration (ng/mL)	Extraction recovery		Matrix effect	
		Accuracy	RSD	Accuracy	RSD
		%	%	%	%
**Plasma**	30	100.1	3.8	108.6	5.6
	400	99.1	3.8	102.1	8.9
	1,600	100.4	9.4	101.0	7.3
**Brain**	15	96.8	6.5	101.0	5.0
	160	94.8	7.0	109.3	7.5
	1,600	95.2	2.2	102.4	4.7
**Heart**	15	101.1	3.1	99.4	7.1
	160	92.5	5.8	100.5	2.1
	1,600	89.0	4.1	110.4	9.5
**Liver**	15	99.2	4.1	103.0	3.5
	160	97.7	3.8	109.6	6.7
	1,600	94.0	5.7	105.0	5.5
**Spleen**	15	99.2	6.3	112.9	6.4
	160	89.4	2.0	105.8	3.0
	1,600	97.8	7.5	112.7	4.0
**Lung**	15	100.0	4.4	113.2	9.6
	160	106.5	1.4	113.5	4.6
	1,600	96.5	3.0	100.1	4.4
**Kidney**	15	100.0	9.7	97.3	9.8
	160	102.6	6.6	94.8	3.1
	1,600	91.7	4.7	90.5	3.1
**Intestine**	30	103.5	5.0	105.2	4.1
	400	98.0	6.1	90.8	5.7
	4,000	106.7	4.2	106.9	4.8
**Colon**	30	100.9	6.0	98.4	6.4
	400	97.6	0.9	106.1	6.7
	4,000	99.9	1.3	91.8	2.6

The stability data of curcumol in rat plasma and tissues are listed in Table 4. The results showed that curcumol was stable for 12 h at room temperature and stored in the autosampler for 12 h. After three freeze-thaw cycles and 30 days at −80°C, no significant degradation was observed.

**TABLE 4:** Stability of curcumol in rat plasma and tissue homogenates (n = 5).

Sample	Spiked concentration (ng/mL)	12 h short-term		Auto-sampler (12 h,-4°)		Three freeze-thaw cycles		Long-term (30days,-80°)	
		Accuracy	Precision	Accuracy	Precision	Accuracy	Precision	Accuracy	Precision
		%	(RSD, %)	%	(RSD, %)	%	(RSD, %)	%	(RSD, %)
**Plasma**	30	104.4	5.1	102.3	9.6	105.2	4.1	108.8	3.1
	400	105.5	4.8	110.3	1.6	110.3	3.3	96.0	8.0
	1,600	91.9	1.3	105.6	3.7	112	1.7	97.9	9.6
**Brain**	15	109.3	4.2	97.2	3.0	98.0	5.7	107.6	1.9
	160	105.3	8.0	101.9	1.8	109.6	2.3	94.9	4.0
	1,600	94.4	2.1	88.0	2.9	86.0	0.7	92.1	4.2
**Heart**	15	107.1	5.4	103.6	4.9	105.7	2.7	103.5	6.7
	160	109.4	1.5	102.5	8.0	99.3	7.5	108.8	1.8
	1,600	90.6	1.5	90.4	4.3	92.0	5.1	112.9	0.6
**Liver**	15	108.1	3.8	104.5	8.6	102.5	3.4	96.5	8.5
	160	109	5.1	110.6	2.4	111.1	1.8	109	2.6
	1,600	89.6	1.6	98.1	1.4	108.6	2.5	94.3	3.5
**Spleen**	15	102.8	9.1	105.1	10.5	105.9	5.6	102.4	6.7
	160	110.5	1.5	109.3	1.2	108.8	1.9	109.6	2.2
	1,600	89.3	1.0	98.6	1.4	97.9	2.1	100.5	0.3
**Lung**	15	106.8	8.2	107.5	6.4	106.0	6.6	108.8	1.7
	160	111.9	2.4	108.5	4.9	104.0	7.3	101.6	7
	1,600	92.3	7.4	93.9	4.9	96.8	2.6	102.4	2.4
**Kidney**	15	109.1	1.7	98.7	8.6	100.7	4.9	99.9	2.2
	160	109.5	0.6	107.6	8	97.3	8.1	103.6	0.9
	1,600	87.9	0.6	102.6	5.7	104.6	4.8	94.9	6.5
**Intestine**	30	108.7	4.8	99.8	3.5	103.8	6.5	94.0	6.7
	400	107.7	2.9	94.2	2.1	89.5	2.8	105.3	2.1
	4,000	93.6	4.1	88.6	1.6	92.3	2.6	91.9	2.1
**Colon**	30	109.5	1.3	97.2	6.5	100.5	6.2	104.8	4.4
	400	109.5	1.0	98.4	1.7	103.2	1.8	101.1	2.3
	4,000	89.9	3.7	92.8	5.2	89.5	1.0	102.3	6.8

### Pharmacokinetic parameters and oral bioavailability in rat

**FIGURE 4:**
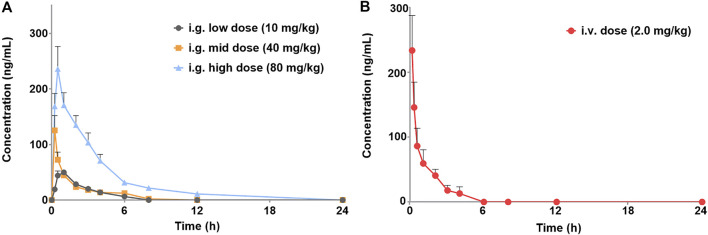
Plasma concentration-time profiles of curcumol are given **(A)** i.g. Doses of 10, 40, 80 mg/kg of curcumol (*n* = 5, mean ± SEM) and given **(B)** i.v. Dose of 2.0 mg/kg of curcumol (*n* = 5, mean ± SEM).

**FIGURE 5:**
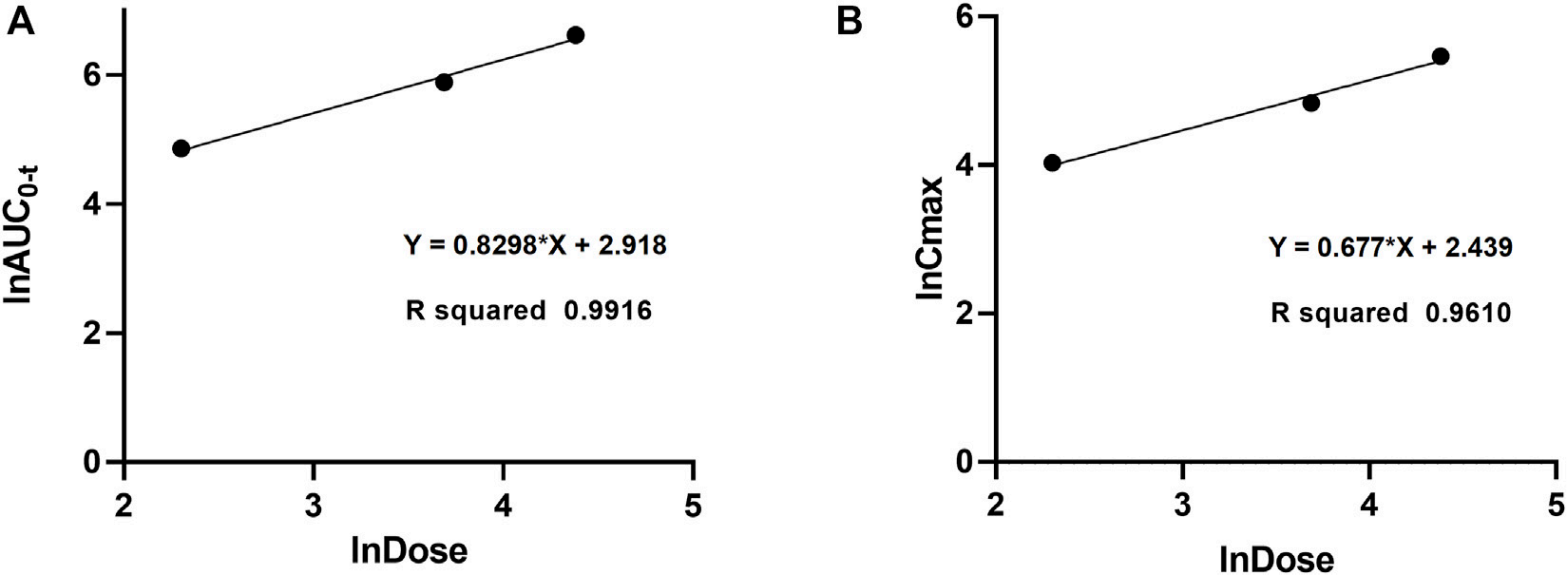
Dose proportionality of AUC_0-t_ **(A)** and C_max_ **(B)** for curcumol in rats at doses of 10, 40, and 80 mg/kg after oral administration.

The validated method for quantifying curcumol was applied to pharmacokinetic studies in rats. The mean plasma concentration-time curves and the main pharmacokinetic parameters of curcumol are shown in Figure 4 and Table 5, respectively. After oral administration of 10, 40, and 80 mg/kg curcumol, the T_max_ was less than 1.0 h, indicating a rapid absorption of curcumol in rats. Quick elimination of curcumol was observed in the plasma with half-lives ranging from 3 to 5 h. Figure 5 shows that the increasing oral dose increased the C_max_ and AUC. After an intravenous injection of 2.0 mg/kg curcumol, the pharmacokinetic results showed that AUC_0−t_ and AUC_0−∞_ were 196.15 ± 40.15 and 218.09 ± 42.97 ng/mL·h. The C_max_ was 233.80 ± 53.73 ng/mL, and the T_1/2_ was 1.25 ± 0.42 h. The apparent V_d_ of curcumol was 17.23 ± 7.24 L/kg, and the CL was 9.45 ± 1.78 L/h/kg. These results indicated a quick elimination of curcumol in plasma and a certain extent of tissue distribution. The absolute oral bioavailability was calculated by comparing the AUCs of curcumol after oral and intravenous administration according to Equation: F (%) = (AUC_ig_ × Dose_iv_)/(AUC_iv_ × Dose_ig_)×100, the absolute bioavailability of curcumol ranges from 9.2% to 13.1% at the three oral doses.

**TABLE 5:** The main pharmacokinetic parameters after oral administration of 10, 40 and 80 mg/kg curcumol; intravenous injection of 2.0 mg/kg curcumol (n = 5, mean ± SD).

Parameters	i.v. (2.0 mg/kg)	i.g. Low dose (10 mg/kg)	i.g. mid dose (40 mg/kg)	i.g. high dose (80 mg/kg)
T_max_(h)	0.08 ± 0.00	0.80 ± 0.27	0.30 ± 0.11	0.45 ± 0.11
C_max_ (ng/mL)	233.80 ± 53.73	56.28 ± 8.35	126.36 ± 58.4	237.60 ± 87.97
AUC_0-t_ (μg/L*h)	196.15 ± 40.15	128.87 ± 11.49	360.18 ± 34.08	746.09 ± 231.26
AUC_0-∞_(μg/L*h)	218.09 ± 42.97	181.76 ± 39.45	431.52 ± 20.6	837.18 ± 218.79
MRT_0-∞_(h)	1.68 ± 0.47	4.29 ± 1.92	4.41 ± 0.65	4.87 ± 1.18
t_1/2_(h)	1.25 ± 0.42	3.04 ± 1.86	3.86 ± 0.86	4.59 ± 1.66

### Tissue distribution

**FIGURE 6:**
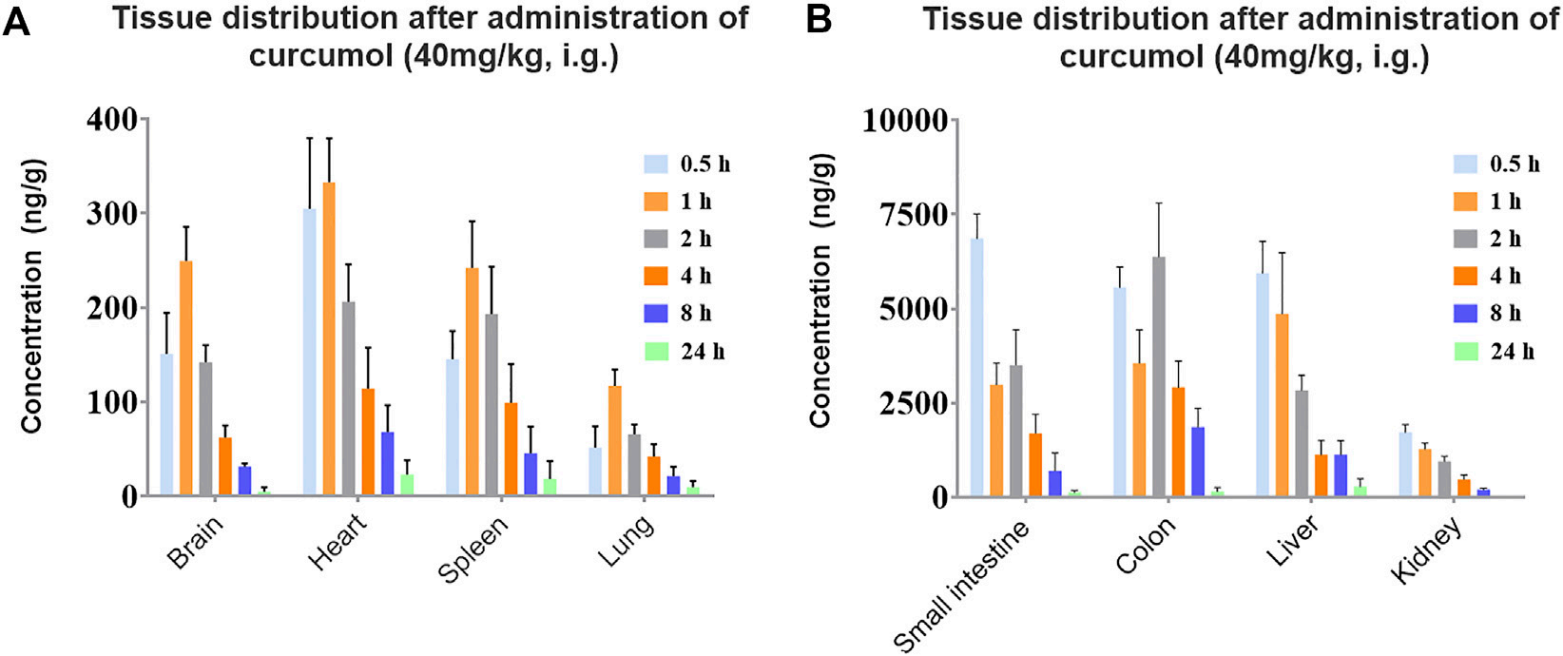
Concentration of curcumol in rat tissues after an oral administration of 40 mg/kg; **(A)** brain, heart, spleen, lung, **(B)** small intestine, colon, liver, kidney. (*n* = 5, mean ± SEM).

To further reveal the characteristics of curcumol *in vivo* and potential target organs, the quantitative method in rat tissues was used to investigate the distribution of curcumol in rat tissues after 40 mg/kg curcumol oral administration. According to the pharmacokinetic study results, six time points of 0.5, 1.0, 2.0, 4.0, 8.0, and 24 h were selected for tissue distribution in this experiment, including the distribution phase, equilibrium phase, and elimination phase. The concentrations of the brain, heart, liver, spleen, kidney, lung, small intestine, and colon within 24 h were shown in Figure 6. In most tissues, the highest concentration of curcumol can be observed within 0.5–1 h, suggesting a rapidly and widely distributed. After 8 h, the tissue concentration decreased obviously with no long-term accumulation. As shown in Figure 6, the Tmax of curcumol in the intestine and colon was 0.5 and 2 h, with the Cmax of 6.86 ± 0.65 and 6.37 ± 1.42 μg/g respectively, which were much higher than that in other tissues.

### *In Vitro* plasma protein binding

A study on plasma protein binding rate was carried out to reveal the distribution of curcumol in blood. We studied protein binding rates at concentrations ranging from 100–600 ng/mL, containing the concentration 126.36 and 237.60 ng/mL, which were the C_max_ of curcumol at the effective dose 40 and 80 mg/kg. The rapid equilibrium dialysis system separated bound and unbound drugs from spiked plasma. Calculate the percentage of the curcumol bound as follows: Unbound (%) = (conc. In buffer chamber/conc. In plasma chamber) × 100%; Bound (%) = 100% - Unbound (%). Plasma protein binding of curcumol was found to be high in rat plasma, with 85.6%, 91.4%, and 93.4% at 100, 300, and 600 ng/mL, respectively.

## Discussion

Previous study has paid much attention to the pharmacological activity of curcumol, particularly its remarkable anti-tumor activity and anti-liver fibrosis. The pharmacological actions of curcumol in different tissues and its wide anti-tumor spectrum indicate its wide systematic distribution. However, few studies have focused on its pharmacokinetics and tissue distribution characteristics. Therefore, to better explore potential target organs and mechanisms of curcumol activity, we studied curcumol pharmacokinetics and tissue distribution in rats.

In our study, a sensitive and efficient UHPLC-MS/MS method was developed to quantify curcumol in rat plasma and tissue samples. Compared with the existing methods, our method is more suitable for analyzing bio-samples due to its simplicity and short analysis time (3 min) (Zhang et al., 2007; Li et al., 2018; Ren et al., 2021; Song et al., 2021). It has been successfully applied to evaluate curcumol as an individual chemical rather than its TCM extract in pharmacokinetic, tissue distribution and plasma binding rate profiles.

To investigate the absorption of curcumol, we measured the concentrations of curcumol in rat plasma at different time intervals. Previous study has reported that the half-life of curcumol in blood was less than 3 h after administration of Rhizome Curcumae extract (Li et al., 2014). Considering its quick elimination, the termination time of 24 h was set, which lasted almost 5-8 half-lives of curcumol. The oral administration doses, 10, 40, and 80 mg/kg, were selected based on the previous pharmacodynamic experiments (Li et al., 2019; Zuo et al., 2020). Due to the low aqueous solubility of curcumol, a relatively low dose of 2.0 mg/kg for intravenous administration was used.

Pharmacokinetic results of curcumol indicated a rapid absorption of curcumol in rats. Moreover, quick elimination in the plasma was observed. These results suggest that curcumol is a small molecular compound directly absorbed through the gastric mucosa with a relatively fast absorption rate. (Wu et al., 2022). In addition, the values of AUCs and C_max_ increased with the three oral doses and exhibited a linear relationship. In this case, we estimate the absolute bioavailability of curcumol, ranging from 9.2% to 13.1%. Curcumol is a high hydrophobic and low aqueous soluble compound. The low aqueous solubility of curcumol may be an important reason for its limited bioavailability resulting in insufficient absorption in the gastrointestinal tract (Chi et al., 2022). A proportion of curcumol is probably excreted in feces without being absorbed into systemic circulation. Furthermore, there may be a first-pass effect, which reduces the amount of curcumol entering the systemic circulation and distributing to other tissues and finally causes a low bioavailability *in vivo*. Further studies on the metabolic stability and excretion patterns of curcumol will help to explain the low bioavailability of curcumol. The results showed a wide distribution of curcumol in most organs and the gastrointestinal tract after a single dose of 40 mg/kg. The concentration trend of curcumol in rat tissues is as follows: $C_{small\;intestine}\approx C_{colon}>C_{liver}>C_{kidney}>C_{heart}>C_{spleen}>C_{brain}>C_{lung}.$

We found that curcumol was mainly enriched in the small intestine and colon. It may be one of the reasons for its remarkable anti-gastroenteric tumor activity (Gao et al., 2021). Except for gastrointestinal tracts, curcumol mostly accumulates in the liver and kidney. The high concentrations of the curcumol in these organs may serve as the foundation to exert its potential pharmacological activities. The concentration of curcumol could be rarely detected 24 h after oral administration, indicating a quick elimination and no apparent retention of curcumol in tissues. In addition, curcumol was detected in the brain, suggesting it can pass through the blood-brain barrier.

The plasma protein binding rate is closely related to the concentration of free drugs in plasma. The study of plasma protein binding rate helps explain drug-drug interaction, pharmacodynamics, and pharmacokinetic characteristics (Xiang et al., 2022). The plasma protein binding rate of curcumol ranged from 85.6% to 93.4% among the three concentrations. The high plasma protein binding of curcumol may be the main reason for its rapid distribution and elimination in rats after oral administration. As the concentration increased, the plasma protein binding rate of curcumol increased slightly, which may due to the plasma protein binding sites not completely saturated within the selected concentration ranges (100–600 ng/mL). Therefore, it could be helpful to further investigate the plasma protein binding of curcumol at higher concentrations. In addition, due to inter-species differences between humans and rats, future study is needed to determine the protein binding rate in human plasma and explore whether the high protein binding rate will affect the drug interaction.

In conclusion, a UHPLC-MS/MS method was established to investigate the pharmacokinetics, tissue distribution, and plasma protein binding of curcumol and this is the first investigation into curcumol as an individual chemical in rats. The developed UHPLC-MS/MS method was validated and successfully applied in the pharmacokinetics and tissue distribution study in rats. Our results showed that curcumol was absorbed and eliminated quickly in rats. The absolute bioavailability of curcumol ranges from 9.2% to 13.1%. Meanwhile, curcumol could be detected in various tissues, indicating a wide distribution of curcumol in rats after oral administration. In addition, a high percent of plasma protein binding ranging from 85.6% to 93.4% of curcumol was found in rat plasma. The current findings provide valuable insight into the pharmacokinetic process and distribution characteristics of curcumol, which could contribute to its further new drug studies and clinical application.

## Data Availability

The original contributions presented in the study are included in the article/Supplementary Material, further inquiries can be directed to the corresponding authors.

## Abbreviations

ADME, absorption, distribution, metabolism, and excretion; ESI, electrospray ionization; FA, formic acid; HPLC, High-performance liquid chromatography; MRM, multiple-reaction monitoring; QC, quality control; %RSD, relative standard deviation; R2, correlation coefficient; SD, standard deviation; SEM, standard error of mean; TCM, Traditional Chinese Medicine; UHPLC-MS/MS, Ultra-performance liquid chromatography with coupled with triple quadrupole mass spectrometry.
